# Preoperative lymphoscintigraphy and triangulated patient body marking are important parts of the sentinel node process in breast cancer

**DOI:** 10.1186/1477-7819-3-56

**Published:** 2005-08-24

**Authors:** Borys R Krynyckyi, Suk Chul Kim, Chun K Kim

**Affiliations:** 1 Department of Radiology, Division of Nuclear Medicine, The Mount Sinai School of Medicine, The Mount Sinai Hospital, New York, New York, USA

**Keywords:** Breast Cancer, Sentinel Lymph Node, Morbidity, Lymphoscintigraphy

## Introduction

Failure to visualize or correctly visualize sentinel nodes (SN) during preoperative lymphoscintigraphy (LS) is a frustrating problem. Most of these instances occur due to inexperience in performing the studies, and can be realized and corrected with the use of proper technique, even in centers that do not have state of art equipment. This is now becoming a major issue, as more and more sentinel node biopsies are being performed, and will increasingly gain more importance once sentinel lymph node biopsy (SLNB) becomes the standard of care in patients with breast cancer world wide.

Pandey et al. [1] has recently reported two unfortunate experiences with LS during SLNB. From the images and the description of the cases, it appears that the studies were suboptimal in both imaging and injection technique, and understandably, completely frustrating to any surgeon.

In the second case, contamination of the patient by stray radioactivity from the perilesional injections is suggested by the authors as the cause of the superior focus that appeared in the supracalvicular region [1]. Actually, true contamination is very easy to realize with proper imaging technique [2-4]. When multiple angled views (0°, 45°, 90°) are obtained, including standing/sitting views and triangulated body marking (TBM), contamination is extremely unlikely to be missed, as it is always *surface *based. In over 2000 LS cases we have performed, the very rare stray activity has always been picked up for what it is, before the patient is presented to the surgeons [2-4].

In the first case, a SN was hidden by the injected perilesional activity [1]. This is a known issue when the primary lesion is located near the axilla itself. Multiple strategies exist for dealing with diffusion and scatter from the injection site hiding the adjacent SNs in the axilla, and are described below:

1) When performing perilesional injections of radiotracer, multiple angled views and standing views, which use gravity to markedly shift the injected activity inferiorly and medially away from the axillary SN compared to the supine position, are indispensable [3,4]. In addition, the perilesional injections should be on the side of the lesion away from the axilla to further lessen diffusion of activity from the injection site from obliterating the SN [3].

2) The volume of perilesional injections should be reduced when injecting close to the axilla to lessen diffusion and inherent scatter of activity (maintain below 3 ml) [3].

3) Upwardly offset energy windows can be used in the camera to reduce "relative" scatter in the images to help better see the SNs [2-4].

4) Proper image display techniques are critical; the images presented for the first case [1] appear to display suboptimal adjustment as related to upper level, contrast/gamma/threshold. Proper adjustment parameters are crucial for the delineation of a faint SN adjacent to intense activity from the injection site [4].

5) Yet, finally, an even better solution to the complexities of upper outer quadrant lesions, is to shift part or all of the injected dose to the areolar region [3-5]. This will largely or completely resolve the issues of injected activity/diffusion/scatter hiding a closely approximated SN in the axilla [3-5]. In addition, An *Adaptive Injection Technique *(AIT) can be employed to allow a limited control over the number of echelon nodes realized, and improve overall SN intensity. High injection volumes (0.8–1.0 ml) administered over 1–2 minutes at the "*areolar-cutaneous junction*", referred to by us as *LymphoBoost *(LB), tend to nearly always delineate SNs (96.6%–100%) [4-6], and often multiple distant echelon nodes [3-7]. This is because these very shallow LB injections are extremely efficient at delivering activity to the SN compared to perilesional intra-parenchymal injections, and even intradermal over the lesion injections [3-7]. Compared to concurrently administered perilesional and intradermal over the lesion injections, LB areolar injections, and other areolar type injections in general, delineate essentially the same primary nodes (excluding internal mammary nodes [3,5,6]) in the vast majority of patients, and do so with several times the efficiency of getting activity into the SN [3-7]. The LB areolar injections occasionally depict additional nodes closer to the injection site, the "*reverse echelon node*" (REN), a feature of areolar type injections in general related to injection location [3,5,8]. They also delineate additional conventional echelon nodes compared to perilesional injections [3,5-7]. There is preliminary evidence that low injection volumes (0.1–0.3 ml) at the areolar-cutaneous junction tend to delineate fewer echelon nodes (which could be viewed as advantageous by some), and also result in fainter nodes, compared to high injection volumes (0.6–1.0 ml) [[5], (data pending publication)]. In addition, low injection volumes are also more prone to failure in delineating the SNs on LS as evidenced by a low 78.5% SN visualization rate during LS in a recent study by Rousseau et al. [9] when using very low volume 0.1 ml injections of radiotracer in a manner similar to LB. Utilizing an AIT, the initial LB injection can be performed using a low or moderate volume of injection, if less echelon nodes are desired, optionally followed by an additional LB injection with a higher volume (0.8–1.0 ml), if needed, when better SN delineation is required (more or brighter SNs). The need for a second injection is based on the viewing of sequential dynamic image sets obtained during the injection process [3-7]. Alternately, only a single high volume (0.8–1.0 ml) LB injection can be performed for the study, as a means to simplify the process.

As judiciously mentioned in the case report by Pandey et al. [1], an additional cause of non-visualization of SNs on LS is extensive replacement of macrophages in the SNs by tumor. This can lead to non-visualization of SNs or SNs that are very faint, if visualized at all [9-11]. However, utilizing the AIT described above with LB injections, will usually maximize the activity in the SNs (many times more than with isolated perilesional injections) making the missed SN on LS images from tumor infiltration (because it is very faint) less likely.

We perform over half of our studies using a two day protocol, as this allows more time for the nuclear medicine department to work with the patient, allows us to take multiple angle and standing views, perform TBM, and eliminates the rush to complete the study and print the images. The two day protocol completely avoids creating any delays in the surgical schedule related to LS imaging and TBM, when surgery is performed the morning after the prior afternoon of imaging. It also allows surgery to be performed at centers remote from the location performing LS and TBM.

The often misunderstood [12-14] and least realized benefits of LS and TBM pertains to their ultimate purposes; i.e. providing a map of the nodes so the surgeons can reduce the amount of dissection performed during SLNB, thereby reducing the morbidity of the procedure of SLNB itself. Even if successful visualization of SNs occurred in only 50% of LS studies, their worth would still be priceless to these women, as a tool to further reduce morbidity during SLNB, compared to the potentially higher levels of morbidity when using only the probe without LS or TBM [15-17].

Fortunately, 94.7%–100% of studies demonstrate SNs when using optimal techniques of areolar injection and various levels of LS image acquisition [4-6,17-21], making these underutilized tools of morbidity reduction applicable to nearly all women undergoing SLNB. This view appears to be in some ways shared by the authors in the case report by Pandey et al. [1], as a triple technique consisting of images, probe and dye is still suggested, regardless of the misfortunate LS events in the two cases presented [1].

In this light, the additional utility of standing/sitting views, with the arm out 90° to the body axis, warrants further explanation. These views are the most accurate for delineating the true number of SNs in the axilla, as they separate bunched SNs that can appear as a cluster (single focus) on supine views [3,4,22-28]. They achieve this by shifting attenuating breast tissues away from the axilla (more SN counts), allowing the camera to be closer to the SNs (better SN resolution), shifting the injected activity away from the axilla (less scatter from injection site to hide SNs) and by stretching out the lymphatic channels from their overlapped/bunched state in the spine position, to their natural drawn out state when the patient is standing (better separation between SNs) [3,4,22-28]. By providing surgeons with a more accurate number and "relative" orientation of SNs in the woman's body, they can better plan on how extensive or selective their dissections are going to be, by more accurately knowing the number of nodes to expect.

Standing/sitting views are naturally not useful for TBM, which requires the patient to closely approximate the surgical position to be meaningful, i.e. triangulation performed on supine anterior and oblique views with the arm out in the 90° surgical position. TBM has been previously described, and can help provide skin references regarding SN location for surgeons before incision [2,3,26,27]. Briefly, the technique consists of the following: with the woman supine, back flat against the imaging table with the arm in the surgical position, an anterior image is obtained and the SN is noted on the monitor and a mark (tape, pen marker, electronic) is made on the monitor screen surface over the SN seen on the monitor. A radioactive point source is brought into the field of view and moved along the woman's chest until it is positioned under the reference point on the monitor surface (the tape or marker point placed earlier on the screen surface while refreshing/updating the image), at which time the woman's body is marked with a indelible color marker. This is repeated in the 45° projection, as lateral views are blocked by the arm in the surgical position. Triangulation into the body along the anterior and 45° projection of the skin surface markings estimates SN location at the crossover point in the body [2,3,16]. The TBM technique can be useful in select patients by shorting the time of the initial probe survey during surgery. It can also help when decay has reduced the counts in the SNs when using two day protocols and in obese patients, where effective directionality and sensitivity of the probe are poor at the skin surface because of attenuation and increased distances from the SNs [2,3,7,27,28]. The technique of TBM will not accurately delineate the SN position when the breast position is different, arm position has shifted, or the patients torso is rotated differently during surgery compared to the position during chest marking during LS. These technical issues must be kept in mind when using the TBM method as an additional guide during the initial probe survey, and an agreed upon routine between surgeon and imaging specialist is required.

None of these techniques require the latest gamma camera equipment, in fact nearly all our studies are performed on cameras at least 12 years old [2-7]. What is required to obtain the best LS images and the most accurate TBM, is a full understanding of the issues and details involved in the techniques of injection, LS imaging and TBM [2-7,10,16,17,24,26-28].

## Abbreviations

SN Sentinel node

LS Lymphoscintigraphy

SLNB Sentinel lymph node biopsy

TBM Triangulated body marking

AIT Adaptive injection technique

LB *LymphoBoost*

## Competing interests

The author(s) declare that they have no competing interests.

## Authors' contributions

BRK Conceived the manuscript.

SCK Assisted with conception, edited manuscript.

CKK Assisted with conception, edited manuscript.
